# Accidental Acetone Ingestion in Liver Transplant Patient With Alcohol Relapse: A Case Report

**DOI:** 10.7759/cureus.32551

**Published:** 2022-12-15

**Authors:** Akash Chakravartty, Prachi B Patel, Pranjal Mishra, Rishav Akilla, Vera Luther

**Affiliations:** 1 Neurology, Wake Forest Baptist Medical Center, Winston Salem, USA; 2 Internal Medicine, Philadelphia College of Osteopathic Medicine, Atlanta, USA; 3 Internal Medicine, Wake Forest Baptist Medical Center, Winston Salem, USA; 4 Research, George Ranch High School, Houston, USA; 5 Internal Medicine, Section on Infectious Diseases, Wake Forest Baptist Medical Center, Winston Salem, USA

**Keywords:** alcoholic pancreatitis, alcoholic cirrhosis, liver transplantation, ketoacidosis, alcohol use disorders, acetone ingestion

## Abstract

Acetone is one of the three main types of ketone bodies that can be found in ketoacidosis, along with acetoacetate, and beta-hydroxybutyrate. Any of these three ketone bodies can be found in the blood after the natural breakdown of fatty acids in diabetes, starvation, or alcoholic ketoacidosis. However, a patient can also develop acetone poisoning from ingestion of common household products such as nail polish removers, paint removers, isopropyl alcohol, or other detergents and cleaners. Ingestion is usually accidental in adults and children and can lead to severe damage to the liver, heart, nervous system, and kidneys. In rare cases, large amounts of ingestion can lead to life-threatening conditions or death. Our case reports a man with a history of alcoholic cirrhosis status post liver transplantation, who unintentionally ingested acetone, mistaking the contents of small bottles for vodka. The patient presented with several syncopal episodes, anion gap metabolic acidosis, transaminitis with hyperbilirubinemia, and pancreatitis.

## Introduction

Acetone is one of the three ketone bodies associated with ketoacidosis, along with acetoacetate and beta-hydroxybutyrate [1]. In healthy individuals, these ketone bodies can be naturally produced in the liver by the breakdown of fatty acids. This mainly occurs in fasting states when glucose stores and insulin levels are low, and glycogen breakdown cannot produce enough glucose [2,3]. The body continues to have normal glucagon and epinephrine levels, causing fatty acids to be released from fat cells. The fatty acids travel to the liver through the blood and are broken down into ketone bodies through a process called ketogenesis [4]. These ketone bodies can then be used by other tissues in the body for adenosine triphosphate (ATP) production through the tricarboxylic acid (TCA) cycle [4]. During the TCA cycle, ketone bodies are broken down to acetyl-CoA, which is formed into oxaloacetate, then glucose. This glucose can be broken down to form ATP. Excessive ketone bodies raise blood sugar levels and often result in hyperglycemia as the body’s natural response to fasting states and starvation.

However, excess ketone bodies in the blood can be problematic as ketone bodies are naturally acidic. The presence of excess ketones leads to metabolic acidosis along with an elevated anion gap as bicarbonate is utilized to offset the potential of hydrogen (pH) shift. Additionally, acetone can only be excreted in exhaled breath (leading to the pathognomonic “fruity” breath odor) or in small amounts in urine, and high levels of exposure can take days to leave the system [5].

Here, we present the case of a 52-year-old man with a history of alcohol use disorder status post liver transplantation who presented with an anion-gap metabolic acidosis and several unexplained episodes of syncope. After further discussion and testing, the patient was found to have unintentionally ingested acetone.

## Case presentation

A 52-year-old male with a past medical history of an alcohol use disorder, subsequent cirrhosis status post liver transplantation 1.5 years prior, on chronic immunosuppression (Tacrolimus extended-release and Mycophenolate Mofetil) who presented with complaints of several syncopal events over the past several weeks. The patient also had a history of hypertension and type 2 diabetes mellitus. The patient had four episodes of loss of consciousness in the previous two days. The patient stated that he experienced a “warm feeling” before losing consciousness but had no other prodromal symptoms. He had one episode of head trauma from these falls. On further questioning, the patient endorsed a recent relapse of alcohol use. He stated that he found four small, “airplane” bottles of vodka at a carwash and drank them. Previously, the patient had been abstinent from alcohol for six months prior to his liver transplantation. In the emergency department, the patient was tachycardic at 127 beats per minute, and tachypneic at 21 breaths per minute, with a normal blood pressure of 102/68 mmHg and oxygen saturation of 98% on room air. He was alert and oriented to person, place, and time. Relevant laboratory data can be found in Table 1. Urinalysis was only remarkable for ketones. The patient was placed on Clinical Institute Withdrawal Assessment for Alcohol (CIWA) protocol for alcohol withdrawal and admitted to the hospital. A computed tomography (CT) scan of the head and chest showed a diffusely enlarged pancreas, and further evaluation with magnetic resonance imaging (MRI) of the abdomen showed no significant abdominal findings besides diffuse pancreatitis with pancreatic pseudocysts in the pancreatic tail.

**Table 1:** Patient laboratory data

Laboratory Test	Value	Reference Range
White Blood Cell Count	8.9x10^3^/µL	4.4 - 11.0x10^3^/µL
Platelet count	64,000/mcL	150,000-450,000/mcL
Bicarbonate	16 mEq/L	22-26 mEq/L
Creatinine	1.68mg/dL	0.7 – 1.3 mg/dL
aspartate transaminase (AST)	124U/L	10-34 U/L
Alanine Transaminase (ALT)	121 U/L	10-130 U/L
Anion Gap	24mEq/L	12-16 mEq/L
Ethanol Level	28mg/dL	0-50mg/dL

The following morning, the patient was altered and only oriented to the person. His neurological exam showed bilateral resting tremors and dysmetria on finger-to-nose testing. An alcohol screen for volatiles was ordered for determining serum levels of methanol, ethylene glycol, and acetone. The patient was found to have elevated levels of acetone at 30 mg/dL (reference range 0-5 mg/dL) in his blood. National poison control was contacted for further recommendations, which included continuing intravenous fluid resuscitation and monitoring anion gap resolution. Throughout the hospitalization stay, the patient began complaining of blurry vision with and without his glasses, which was not present prior to admission. MRI of the brain without contrast showed age-advanced parenchymal volume loss without any lobar predominance, but no acute intracranial abnormalities. Further evaluation for syncopal episodes showed no evidence of orthostatic, cardiac or other neurological abnormalities.

The patient was eventually discharged after the resolution of his anion gap, acute kidney injury, thrombocytopenia, and transaminitis. However, the patient did not have resolution of his blurry vision, bilateral resting tremors, or upper extremity dysmetria. The patient was discharged with outpatient follow-up appointments with gastroenterology physicians for his pancreatic pseudocysts and with addiction medicine for a recent relapse. The patient was re-admitted one month later for abdominal pain, found to be secondary to necrosis of the pancreatic head. The patient did not require any surgical intervention or antibiotics at that time and was discharged four days later following aggressive IV fluid administration and pain management with opioids.

## Discussion

Acetone ingestion has no defining clinical presentation. As such, a patient’s history of accidental or intentional ingestion is critical in guiding clinicians to include this in their differential diagnosis. As illustrated by this case, patients may be unaware of the ingestion, so maintaining a high index of suspicion and a low threshold for ordering alcohol screens for volatiles may be beneficial when there is a concern for ingestion or a history of alcohol abuse and symptoms that cannot be explained by initial diagnostic testing.

Although acetone ingestion rarely causes death, it can cause permanent damage to a number of organs [3,6]. Case reports and animal models show syncope as a common side effect of acetone ingestion, even days after the initial exposure [5]. This was seen in our patient who had numerous unexplained episodes of syncope following his acetone ingestion. Studies have shown that workers exposed to acetone over long periods of time can develop severe neurotoxic effects, including permanent mood changes, irritability, sleep disturbances, headaches, and neuropathy [6,7]. Nerve conduction studies in the median, ulnar, and peroneal nerves showed statistically significant differences in proximal and distal nerve conduction amplitude, distal nerve latency and velocity in acetone-exposed subjects compared to controls [6].

Additionally, acetone ingestion can be nephrotoxic. Our patient was admitted with a mild elevation in serum creatinine of 1.68 mg/dL (reference range 0.7-1.3 mg/dL) and a blood urea nitrogen (BUN) of 25mg/dL (reference range 6-24 mg/dL). Kidney damage in acetone ingestion is hypothesized to be caused by hypoperfusion from acetone-induced hypotension or arrhythmias, or thorough direct damage to the nephrons [6]. Our patient’s BUN/Cr ratio was 14.8 (reference range 10-21), suggesting intrinsic renal damage through acetone nephrotoxicity. Animal models have shown female rats to have decreased kidney weight from small doses of acetone, while male rats have more histopathological changes from small doses [6]. Case reports have described renal insufficiency and sometimes permanent renal failure in humans, however, epidemiological studies have failed to show consistent, long-term effects.

Treatment of acetone ingestion is almost always supportive. Large amounts of acetone ingestion have been shown to cause respiratory depression and hypotension, so stabilization of breathing, airway, and circulation is critical in these cases [7]. Large-volume intravenous fluid resuscitation can be given for hypotensive patients, and in rare cases, vasopressors may be necessary to help maintain blood pressure. In critically ill patients, hemodialysis can be considered because it has been shown to help in severe cases of acetone poisoning [8]. Unfortunately, acetone is rapidly absorbed in the stomach following ingestion, so induced emesis or gastric lavage is not helpful unless the patient presents within one hour of ingestion [7]. Finally, unlike methanol or ethylene glycol poisoning, 100% ethanol cannot be used as a treatment for acetone poisoning, as ethanol can be broken down into acetone and lead to worsening of symptoms [7].

## Conclusions

Acetone ingestion is an important consideration to keep on the differential diagnosis in the case of unexplained anion gap metabolic acidosis with hyperglycemia and ketones present. A careful history must be obtained, and a low threshold must be kept to obtain serum testing for levels of acetone or other volatile alcohol-based substances if acetone ingestion is suspected.

## References

[1] Ghimire P, Dhamoon AS (2022). Ketoacidosis. https://www.ncbi.nlm.nih.gov/books/NBK534848/.

[2] Akgul Kalkan E, Sahiner M, Ulker Cakir D, Alpaslan D, Yilmaz S (2016). Quantitative clinical diagnostic analysis of acetone in human blood by HPLC: a metabolomic search for acetone as indicator. J Anal Methods Chem.

[3] Umeh C, Gupta RC, Gupta R (2021). Acetone ingestion resulting in cardiac arrest and death. Cureus.

[4] (2022). Ketones: Diabetes Education Online. https://dtc.ucsf.edu/types-of-diabetes/type2/understanding-type-2-diabetes/how-the-body-processes-sugar/ketones/#:~:text=Ketones%20and%20ketoacids%20are%20alternative,supply%20the%20body's%20fuel%20needs.

[5] (2022). Toxicological profile for acetone. https://www.atsdr.cdc.gov/toxprofiles/tp21.pdf.

[6] Mitan E, Callender T, Orha B (1997). Neurotoxicity associated with occupational exposure to acetone, methyl ethyl ketone, and cyclohexanone. Environment Res.

[7] Kumarvel V, Da Fonseca J (2022). Acetone poisoning - a diagnostic dilemma. Eur J Anaesthesiol.

[8] Bradberry S (2011). Acetone. Specific substances.

